# Determination of Personalized IOL-Constants for the Haigis Formula under Consideration of Measurement Precision

**DOI:** 10.1371/journal.pone.0158988

**Published:** 2016-07-08

**Authors:** Simon Schröder, Christina Leydolt, Rupert Menapace, Timo Eppig, Achim Langenbucher

**Affiliations:** 1 Institute of Experimental Ophthalmology, Saarland University, Homburg, Germany; 2 University Eye Clinic, Vienna, Austria; University of Florence, ITALY

## Abstract

The capabilities of a weighted least squares approach for the optimization of the intraocular lens (IOL) constants for the Haigis formula are studied in comparison to an ordinary least squares approach. The weights are set to the inverse variances of the effective optical anterior chamber depth. The effect of random measurement noise is simulated 100000 times using data from *N* = 69 cataract patients and the measurement uncertainty of two different biometers. A second, independent data set (*N* = 33) is used to show the differences that can be expected between both methods. The weighted least squares formalism reduces the effect of measurement error on the final constants. In more than 64% it will result in a better approximation, if the measurement errors are estimated correctly. The IOL constants can be calculated with higher precision using the weighted least squares method.

## Introduction

Cataract is the major cause of blindness world wide [1]. It is characterized by a clouding of the natural human lens. Its treatment requires surgical removal of the lens. The lens can then be replaced by an artificial intraocular lens (IOL). Proper preoperative calculation of the refractive power of the IOL is important in order to achieve the desired visual outcome. State of the art IOL calculation requires at least three parameters: the axial length of the eye, the refractive power of the cornea and an estimation of the IOL position after surgery [2–4]. If a distinct refractive outcome is desired, the target refraction will also be required as a parameter. The Haigis formula requires the distance between the corneal epithelium and the natural lens (the phakic anterior chamber depth) as an additional parameter [5, 6].

The estimation of the postoperative IOL position based on preoperative biometry (refractive power of the cornea, axial length, anterior chamber depth) is a major source of error in the calculation of IOL power [7]. Several formulae can be used to estimate the appropriate IOL power [2–5, 8, 9]. The design parameters provided by the manufacturers (lens constants) allow for an initial estimation of the best suited IOL power, but have to be refined in order to reduce the deviations between planned and achieved post-surgical refraction [10, 11]. These lens constants are optimized according to the postoperative outcomes observed with a specific IOL, in order to minimize the numerical errors for the sample.

Some measurements may include larger measurement errors than others. Measurements that have larger errors should contribute less to the refinement of the parameters than those with smaller errors. The statistical measurement uncertainty results from natural variations in the shape of the eye, uncontrolled lens accommodation and pupil size, variations in the axis of fixation during data acquisition, and measurement resolution [12]. The uncertainty of the axial length *L* measurement contributes approximately 2.8m^−1^ mm^−1^ (additional refractive power in m^−1^ at spectacle plane per measurement error in mm), and the uncertainty of the anterior chamber depth *ACD* measurements −1.4m^−1^ mm^−1^ to the error of the IOL power calculation [7]. Measurement uncertainty is significantly reduced through the proper use of low coherence interferometry for the measurement of the axial length [7, 13, 14]. Nevertheless, it can be beneficial to prepare the data by adequate screening [9] for outliers originating from erroneous data transfer or inaccurate measurements. An approximation method that takes the probability-distribution of measurement errors into account such as a maximum likelihood approach might also increase the predictive potential of personalized IOL-constants.

The Haigis formula [5, 6] is based on a simplified thin lens model of the cornea using only the keratometry values of the anterior cornea to calculate the effective corneal refractive power *K*_*m*_ defined as the average over both keratometry measurements using the keratometer index of *n*_*c*_ = 1.332. The postoperative optical anterior chamber depth *d* does not necessarily correspond to the physiological post-surgical anterior chamber depth. It is defined as the parameter *d* in the the prediction of the lens power *D* [5, 6]

$$\begin{array}{c} D=\frac{n}{L-d}-\frac{n}{\frac{n}{K_{m}+\frac{R_{x}}{1-R_{x}\cdot 0.012\;m}}-d} \end{array}$$ (1)

that on average results in the smallest difference between the planned refraction *R*_*x*_ and the achieved post surgical refractive outcome *F*. The index of refraction of the aqueous humour is accepted as *n* = 1.336 [15]. The phakic anterior chamber depth *ACD* and the axial length *L* are used to predict the postoperative anterior chamber depth *d* of the thin lens calculation (Eq 1).

The optical anterior chamber depth *d* can be estimated with the Haigis formula [5, 6] given by

$$\begin{array}{c} d=a_{0}+ACD\cdot a_{1}+L\cdot a_{2}. \end{array}$$ (2)

The constants *a*_0_, *a*_1_, and *a*_2_ in the Haigis formula characterize each IOL-type. The postoperative optical anterior chamber depth can be derived by solving Eq 1 for *d* given the post surgical refractive outcome *F*. The planned refraction *R*_*x*_ in Eq 1 is replaced by *F*. Substitution of the effective refractive power

$$\begin{array}{c} z=K_{m}+\frac{F}{1-F\cdot 0.012\;m}, \end{array}$$ (3)

allows to write the solution of Eq 1 in the simple form

$$\begin{array}{c} d=\frac{1}{2}\left(L+\frac{n}{z}\right)-\sqrt{\frac{1}{4}{\left(L+\frac{n}{z}\right)}^{2}-\left(L\frac{n}{z}-\frac{n^{2}}{Dz}+n\frac{L}{D}\right)}. \end{array}$$ (4)

In clinical practice the effective optical anterior chamber depth *d* is calculated from Eq 4 for a large number of patients and the corrected IOL-constants for the Haigis formula are calculated by ordinary linear least squares fitting to Eq 2.

The ordinary least squares fitting procedure is sensitive to outliers and does not take into account the measurement uncertainties for *L*, *ACD*, *K*_*m*_, *D*, or *F*. In order to improve the robustness of the optimization of lens constants against measurement uncertainty, we propose to use a weighted least squares method. In this paper, the weighted least squares method is tested and compared to the ordinary least squares approximation with the help of simulated measurement noise for two different biometry devices. The method is demonstrated for the optimization of the IOL constants for the Haigis formula, but can be applied to other IOL power formulae as well.

## Materials and Methods

The ordinary least squares method searches for the minimum of

$$\begin{array}{c} \chi_{\text{olsq}}^{2}=\sum_{i}{\left(a_{0}+ACD_{i}\cdot a_{1}+L_{i}\cdot a_{2}-d_{i}\right)}^{2}. \end{array}$$ (5)

The values of *ACD*_*i*_, *L*_*i*_, and *d*_*i*_ are subject to measurement noise. In a weighted least squares method, the values with higher uncertainties are given smaller weights by multiplying Eq 5 with the reciprocal of the variances $\sigma_{i}^{2}$

$$\begin{array}{c} \chi_{\text{wlsq}}^{2}=\sum_{i}\frac{{\left(a_{0}+ACD_{i}\cdot a_{1}+L_{i}\cdot a_{2}-d_{i}\right)}^{2}}{\sigma_{i}^{2}}. \end{array}$$ (6)

The variances can be calculated by error-propagation

$$\begin{array}{c} \sigma_{i}^{2}={\left(\frac{\partial d_{i}}{\partial L}\sigma_{L}\right)}^{2}+{\left(\frac{\partial d_{i}}{\partial D}\sigma_{D}\right)}^{2}+{\left(\frac{\partial d_{i}}{\partial z}\sigma_{z}\right)}^{2}+a_{1}^{2}\sigma_{ACD_{i}}^{2}+a_{2}^{2}\sigma_{L_{i}}^{2}. \end{array}$$ (7)

The ordinary least square solution that minimizes $\chi_{\text{olsq}}^{2}$ (Eq 5) is used as an estimate for the IOL-constants in the calculation of the variances.

**Fig 1:**
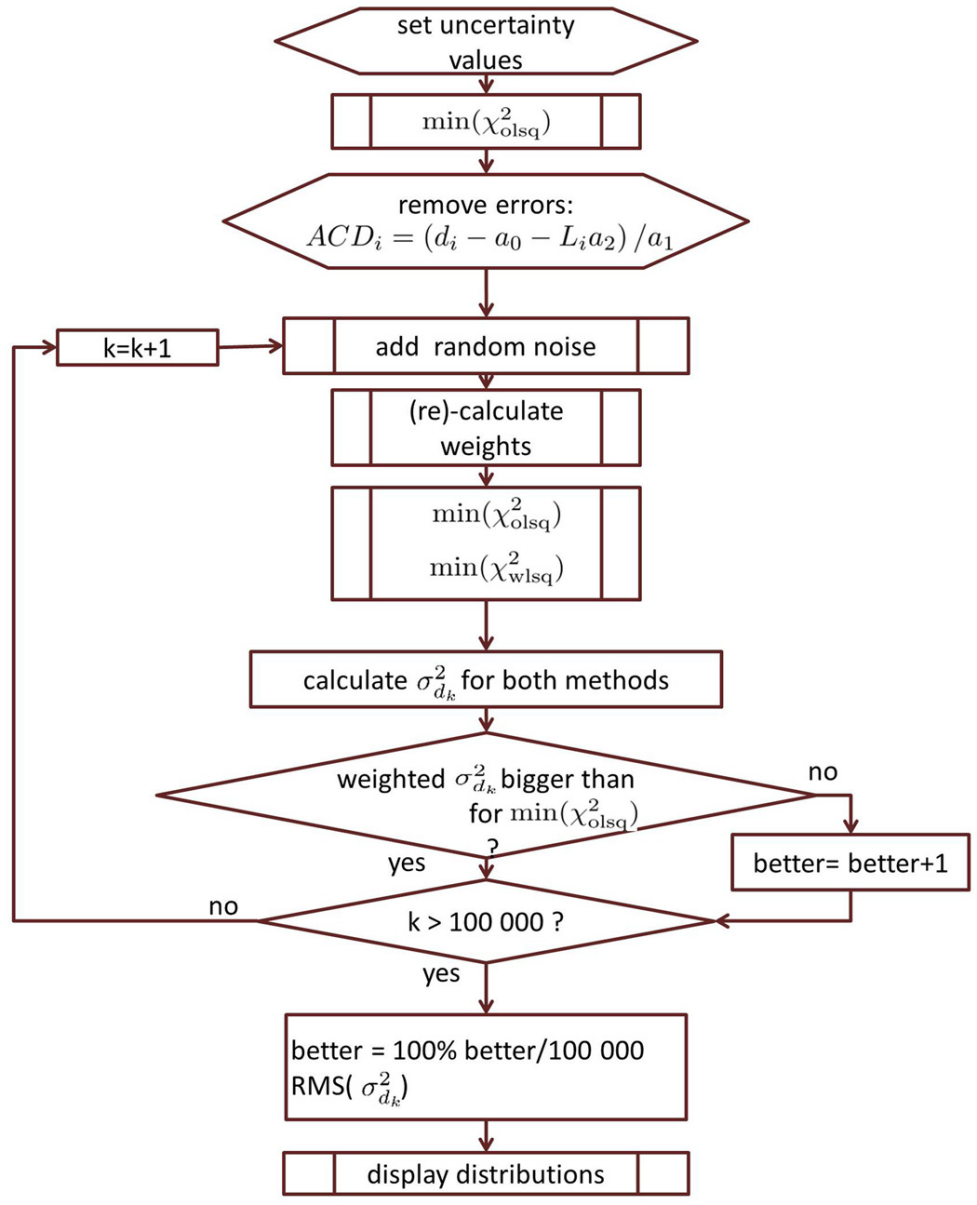
The flow chart for evaluation the robustness of the weighted least squares approximation against measurement noise.

The performance of the approximation model can be tested with the help of simulated measurement noise. The program work flow is illustrated in Fig 1: An ordinary least-squares approximation (Eq 5) is performed on a real data-set. An artificial system of realistic measurements is obtained by replacing the *ACD*_*i*_ values with

$$\begin{array}{c} ACD_{i}=\frac{d_{i}-a_{0}-a_{2}\cdot L_{i}}{a_{1}} \end{array}$$ (8)

using the expression for *d*_*i*_ given in Eq 4. The original ordinary least-squares approximation is thus the ideal solution for this artificial system and has no approximation error. Then, noise with a Gaussian distribution whose width is given by the corresponding statistical uncertainty of each measurement is added onto each of the terms *ACD*_*i*_, *L*_*i*_, *D*_*i*_, $K_{m}^{i}$, and *F*_*i*_. The effective optical anterior chamber depths *d*_*i*_ are recalculated with these perturbed values according to Eq 4. The approximation of the IOL constants for the Haigis formula by the ordinary (Eq 5) and the weighted least squares (Eq 6) method is conducted 100000 times. Each time the result is affected by the random noise and one method may achieve better results than the other. The comparison of both methods therefore requires many repetitions.

One method can be considered more effective than the other when its results deviate less from the ideal values in more than half of all 100000 cases. This means that the variances

$$\begin{array}{c} \sigma_{d_{k}}^{2}=\sum_{i}\frac{{\left(d_{i}-a_{0}^{k}-a_{1}^{k}\cdot ACD_{i}-a_{2}^{k}\cdot L\right)}^{2}}{N}, \end{array}$$ (9)

where the $a_{0}^{k}$, $a_{1}^{k}$, $a_{2}^{k}$ refer to the solution of the perturbed system, are smaller. Another important measure is the square-root of the mean of Eq 9 over all iterations *k*. Robust fitting reveals itself in a narrow distribution of constants $a_{0}^{k}$, $a_{1}^{k}$, $a_{2}^{k}$ (that are not constant in this case, but can vary each iteration *k*).

These simulations are performed using the repeatability values for two different biometers; the IOLMaster 700 (Carl Zeiss Meditec AG, Jena, Germany) and the Aladdin (Topcon Corp., Tokio, Japan). The repeatability for the Zeiss instrument was studied by Kunert et al. [16] and Srivannaboon et al. [17]. The weighted averages of both values according to the number of study-participants are used in this study. They are *σ*_*ACD*_ = 9.4 *μ*m, *σ*_*L*_ = 10 *μ*m, and *σ*_*K*_*m*__ = 0.11 m^−1^. The values for the Topcon instrument were derived by Huang et al. [18]. The average values of the measurements for the cataract group are used here: *σ*_*L*_ = 20 *μ*m, *σ*_*ACD*_ = 45 *μ*m, and *σ*_*K*_*m*__ = 0.11 m^−1^. The statistical uncertainty for the assessment of the final refraction *F* is assumed to be *σ*_*F*_ = 0.2 m^−1^. In accordance with the principles of modern quality assurance, the tolerance interval for the IOL power should be 6 standard deviations [19]. The width of the Gaussian noise *σ*_*D*_ for IOL-power *D* is thus set to 1/6^th^ of the total tolerance interval defined by the International Organization for Standardization (ISO) [20] which depends on the absolute value of *D* (Table 1).

**Table 1:** The standard deviations *σ*_*D*_ of the IOL power as a function of the intervals *D*_min_ < *D* ≤ *D*_max_ where the IOL power *D* can be found. It is set to 1/6^th^ of the total tolerance interval defined by ISO [20].

*D*_min_/m^−1^	*D*_max_/m^−1^	*σ*_*D*_/m^−1^
0	15	0.1
15	25	$\frac{4}{30}$
25	30	$\frac{5}{30}$
30	∞	$\frac{1}{3}$

This is a collaborative work between Saarland University and the University Hospital in Vienna (AKH Wien). The data sets for this study were recorded routinely from patients before and after cataract surgery at AKH Wien by Leydolt and Menapace using the Zeiss IOLMaster 700 device. All data were anonymized and de-identified before they were sent to Saarland University for analysis. Since this is a retrospective study of routinely recorded data, no ethics committee approval was required.

The simulations were performed using *N* = 69 measurements of the EyeCeeOne NS-60YG lens (Croma Pharma GmbH, Leobendorf, Austria). Finally, the method was applied to an independent data set consisting of *N* = 33 measurements on patients that had the Acrysof SN60WF (Alcon Inc., Ft. Worth, TX, USA) implanted. This is intended to show the differences that can be expected between both methods. The refraction was measured six months postoperatively. Mean values and standard deviations of the relevant measurements in both data sets are shown in Tables 2 and 3.

**Table 2:** The number of right N(OD) and left N(OS) eyes included in the calculations, mean keratometer readings and standard deviation for both lens types.

lens	N(OD)	N(OS)	*K*_1_/m^−1^	*K*_2_/m^−1^	*K*_*m*_/m^−1^
NS-60YG	33	36	43.0 ± 1.7	43.9 ± 1.8	43.5 ± 1.7
SN60WF	17	16	43.3 ± 1.7	44.2 ± 1.6	43.7 ± 1.6

**Table 3:** The mean and standard deviation of preoperative anterior chamber depth *ACD*, axial length *L*, lens power *D* and final refraction *F* for both lens types.

lens	*ACD*/mm	*L*/mm	*D*/m^−1^	*F*/m^−1^
NS-60YG	3.09 ± 0.44	23.57 ± 1.4	21.8 ± 3.5	−0.43 ± 1.04
SN60WF	2.95 ± 0.27	23.00 ± 0.66	22.6 ± 2.3	−0.67 ± 0.77

## Results

The first data set contains data of patients which having an EyeCeeOne NS-60Y implant. It comprises *N* = 69 valid equations. The original ordinary least squares approximation results for the IOL-constants is the ideal solution given in Table 4.

**Table 4:** Mean and standard deviation of the IOL-constants for the Haigis formula calculated with the ordinary least squares (OLS) and weighted least squares (WLS) method.

	*a*_0_/mm	*a*_1_	*a*_2_
Ideal	1.168	0.3172	0.1403
**Aladdin**			
OLS	1.155 ± 0.470	0.3168 ± 0.0200	0.1410 ± 0.0205
WLS	1.152 ± 0.424	0.3167 ± 0.0184	0.1412 ± 0.0182
**IOLMaster**			
OLS	1.162 ± 0.464	0.3172 ± 0.0197	0.1406 ± 0.0202
WLS	1.161 ± 0.407	0.3172 ±0.0181	0.1407 ± 0.0178

**Fig 2:**
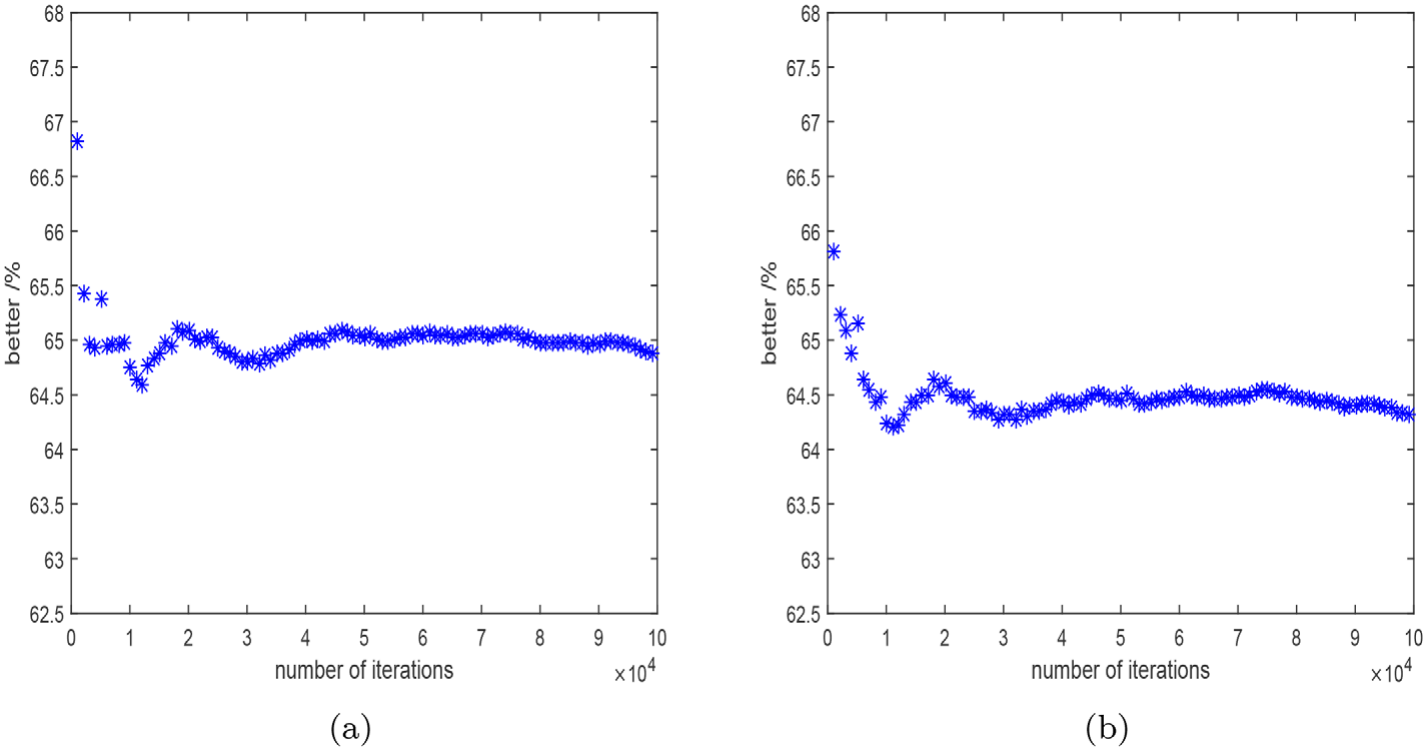
The percentage of cases in which the weighted least squares is superior to the ordinary least squares method as a function of the number of iterations using the repeatability values of the IOLMaster 700 (a) and the Aladdin (b) biometer.

**Fig 3:**
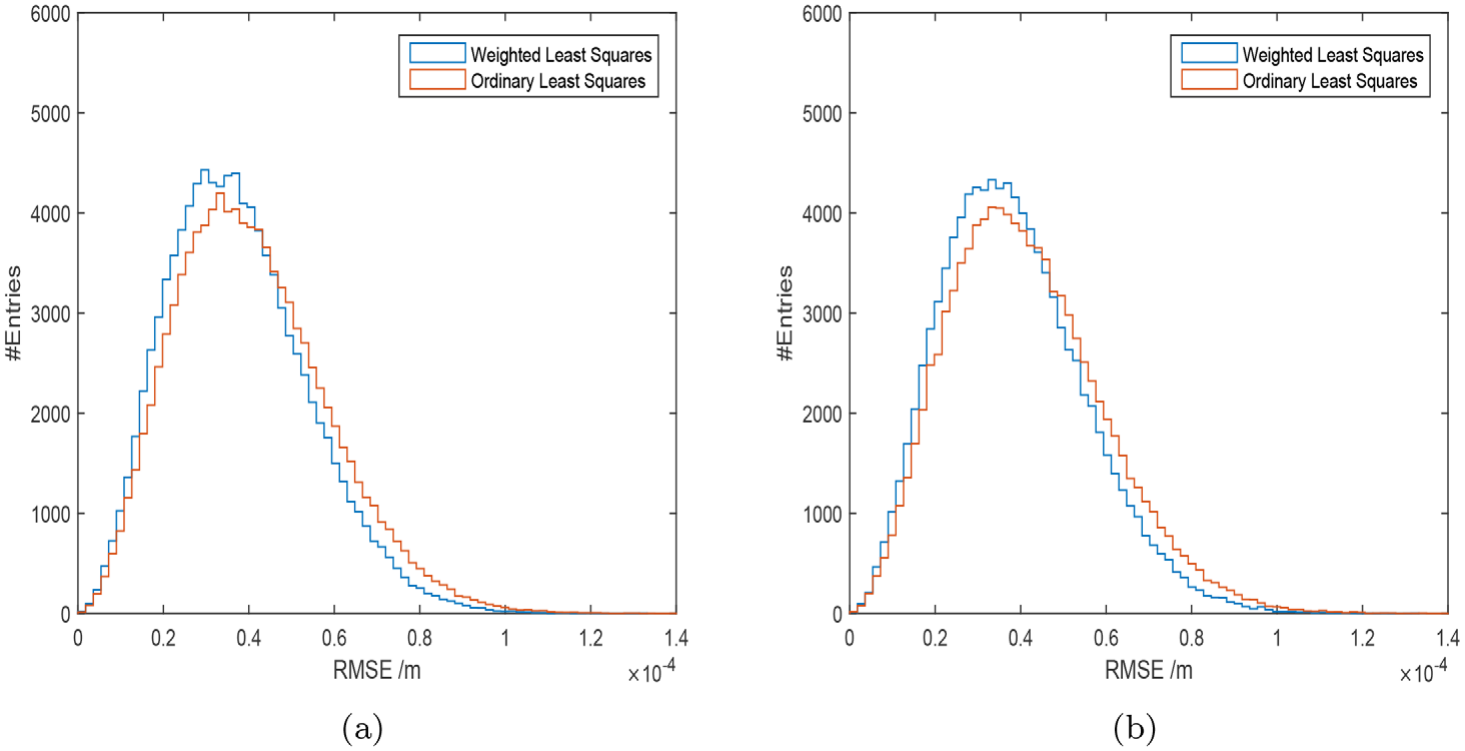
The distribution of the approximation error given by the square root of the mean of Eq 9 using the repeatability values of the IOLMaster 700 (a) and the Aladdin (b) biometer. The values for the weighted least squares approach are shown in blue, the distribution for the ordinary least squares in red.

**Fig 4:**
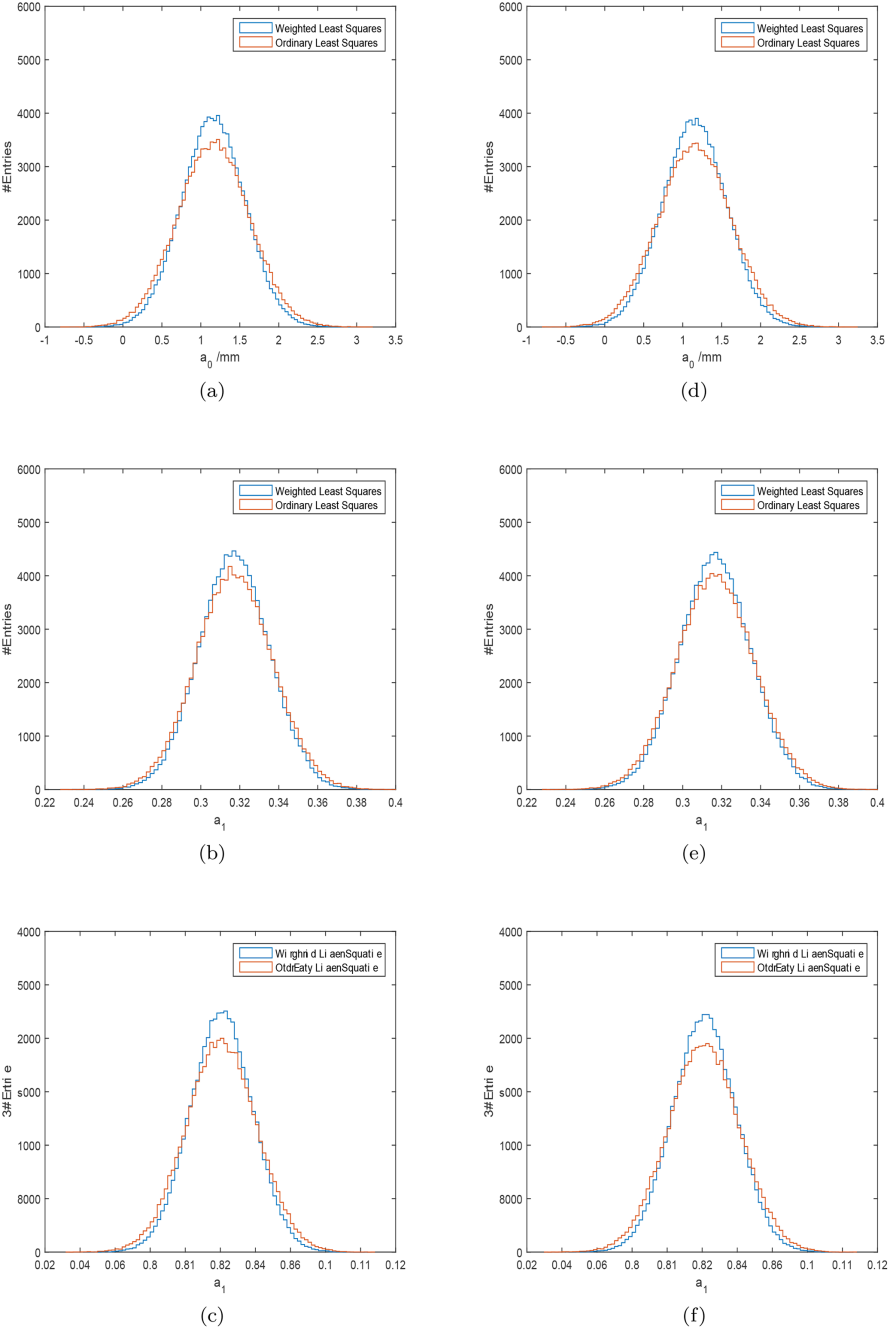
The distribution of the IOL-constants for the Haigis formula *a*_0_, *a*_1_, *a*_2_ calculated with the weighted least squares (blue) and ordinary least squares approach (red). Subfigures (a)–(c) are based on the repeatability values of the IOLMaster 700, subfigure (d)–(f) are based on the repeatability values of the Aladdin device.

Firstly, the uncertainty values of the IOLMaster 700 were used. In 64.9% of all 100000 cases the weighted least squares procedure proved to be superior to the ordinary least squares fitting procedure according to Eq 9. The development of this ratio with iteration number is shown in Fig 2a. The root mean square error (*RMSE*) given by the square-root of the mean of the 100000 $\sigma_{d_{k}}^{2}$ given by Eq 9 is *RMSE*_wlsq_ = 40.97 *μ*m ± 0.05 *μ*m, which is better than the *RMSE* value for an ordinary least squares approach *RMSE*_olsq_ = 44.34 *μ*m ± 0.06 *μ*m. The *RMSE* fitting error of the weighted fit approach is smaller, and its distribution (Fig 3a) has a smaller width compared with the ordinary least squares fitting approach. The median value of the square-root of the $\sigma_{d_{k}}^{2}$ is 36.0 *μ*m with the weighted least squares, and 38.8*μ*m with the ordinary least square method. The distributions of the IOL-constants for the Haigis formula with the weighted approach have smaller widths than those calculated with the ordinary least squares method (Fig 4). The mean and standard deviation of the calculated constants are shown in Table 4.

The calculations are repeated using the uncertainty values of the Aladdin. In 64.3% of all 100000 cases the weighted least squares procedure proved to be superior to ordinary least squares fitting. The development of this ratio with iteration number is shown in Fig 2b. The RMSE errors are *RMSE*_wlsq_ = 41.72 *μ*m ± 0.056 *μ*m, and *RMSE*_olsq_ = 45.03 *μ*m ± 0.061 *μ*m for the weighted and ordinary least squares method respectively. The *RMSE* fitting error of the weighted fit approach is smaller, and its distribution (Fig 3b) has a smaller width compared with the ordinary least squares fitting approach. The corresponding median values of the square-root of the $\sigma_{c_{k}}^{2}$ are 36.7 *μ*m for the weighted fits and 39.4 *μ*m for the ordinary least squares fits. The distributions of the IOL-constants for the Haigis formula with the weighted approach have smaller widths than those calculated with the ordinary least squares method (Fig 4). The mean and standard deviation of the calculated constants are shown in Table 4.

The second data set comprises 33 valid equations. The resulting IOL-constants are shown in Table 5 together with their corresponding weighted RMSE (wRMSE) values given by the root mean square of (*d*_*i*_ − *a*_0_ − *a*_1_ ⋅ *ACD*_*i*_ − *a*_2_ ⋅ *L*_*i*_)/*σ*_*i*_. The uncertainty *σ*_*z*_ of the effective refractive power *z* makes the greatest contribution to the weights $\sigma_{i}^{-2}$ (Eq 7). The mean value and standard deviation of each contribution are shown in Table 6.

**Table 5:** The approximation solutions for the Acrysof SN60WF IOL and their weighted RMSE calculated with the ordinary least squares (OLS) and weighted least squares (WLS) methods.

	*a*_0_/mm	*a*_1_	*a*_2_	wRMSE
OLS	1.90 ± 3.1	0.80 ± 0.34	0.027 ± 0.14	0.8863
WLS	1.69 ± 3.3	0.82 ± 0.36	0.034 ± 0.15	0.8861

**Table 6:** The average contributions to the variances $\sigma_{i}^{2}$ (Eq 7) and their standard deviation as observed for the Acrysof SN60WF IOL, in mm^2^.

${\left(\frac{\partial d}{\partial L}\sigma_{L}\right)}^{2}$	${\left(\frac{\partial d}{\partial D}\sigma_{D}\right)}^{2}$	${\left(\frac{\partial d}{\partial z}\sigma_{z}\right)}^{2}$	$a_{1}^{2}\sigma_{ACD}^{2}$	$a_{2}^{2}\sigma_{L}^{2}$
(2.57 ± 0.26)10^−4^	(2.50 ± 0.52)10^−3^	0.321 ± 0.048	5.63 ⋅ 10^−5^	7.32 ⋅ 10^−8^

## Discussion

The weighted least squares approximation method provided superior results in most cases compared with the ordinary least squares method. More than 64% of its results produce smaller deviations from data that have been cleaned from measurement error than the ordinary least squares method, and the distributions of the IOL-constants for the Haigis formula show smaller widths. Thus, the weighted least squares approximation method delivers results with higher precision than the ordinary least squares method. This is expected as the weighted fit reduces the influence of data with higher uncertainty, and the uncertainties were set correspondingly. This holds true for the uncertainty values of both the Aladdin and the IOLMaster biometer. The results are not limited to these lens types or biometers. Similar improvements can be expected for other IOLs and biometers.

A data set consisting of 69 valid equations was used in order to simulated the influence of Gaussian measurement noise on the approximation. Usually, the IOL constants are optimized on a larger data basis. The data set provides realistic values which were modified before each of the 100000 iterations. The height number of iteration enables us to report very precise results (< 0.1%) concerning which method is superior for the eyes in this data set. Their statistical data is shown in Tables 2 and 3. The differences in the resulting constants between both methods tested on an independent second data set are small compared to the statistical error. The wRMSE value of the weighted least squares approach is smaller for the weighted least squares method. Bigger sample size is required in order to study possible differences in the resulting constants.

In applying this method to calculate the personalized IOL constants it is important that the statistical uncertainties of axial length, anterior chamber depth, corneal refraction, postoperative spherical equivalent, and IOL-power measurements are estimated. The uncertainties of the biometer have only minor influence. This can be seen when the distributions of the Gaussian noise is set according to the uncertainties of one biometer and the weights according to those of another. For comparison, we used data of the EyeCeeOne NS-60YG lens, and set the distributions of the Gaussian noise according to the statistical measurement uncertainties of the Aladdin, but the weights according to the statistical measurement uncertainties of the IOLMaster. The results were identical with those obtained using the statistical uncertainties of the Aladdin for both the Gaussian noise and the calculation of the weights. The weighted least squares method is superior in 64.3% of all cases. Consequently, the weighted least squares approximation approach can be helpful even when the exact values of the statistical uncertainties are unknown. The uncertainty is clearly dominated by the contribution of the effective corneal power *z* (Eq 3). The statistical uncertainty of the measurement of the postoperative refraction *σ*_*F*_ has the strongest influence.

The statistical uncertainties of the measurements are assumed to have Gaussian distributions. The weighted least squares approximation method performs well in the presence of Gaussian noise. However, in the presence of outliers the weighted least squares method might be improved by eliminating them prior to the fit or implementing a robust approximation method that might include a more sophisticated description of the measurement uncertainties, for example by applying lower weights to particularly long eyes. A more realistic description of the uncertainties of the refractive power of the lens *D*, or the uncertainties of the pseudophakic refraction *F*, might improve the performance of the weighted least squares approximation on real data.

## Conclusion

An ordinary and a weighted least squares approach for the refinement of the IOL-constants for the Haigis formula were compared. The differences in the resulting constants are small. However, using a weighted fitting method offers superior robustness against random measurement error and should be used with the correct uncertainties. If the uncertainties are estimated correctly the weighted least squares approximation will have a probability higher than 64% of delivering superior results compared to ordinary least squares fitting. Further studies could test the weighted least square approach for larger data sets and/or different formulae. The procedure can be extended by applying more sophisticated models for the statistical measurement uncertainties. The dominant contribution to the statistical uncertainty in this approximation model originated from the statistical uncertainty of the postoperative refraction measurement.

## Supporting Information

S1 File EyeCeeOne data. The biometric information for patients who got the EyeCeeOne NS-60YG implanted. Right eyes are indicated with OD, left eyes with OS. The table shows the steep and flat keratometry values (D1, D2) their angles, the anterior chamber depth (ACD), the power of the implanted IOL (IOL power), target refraction and the refraction six month after surgery. Visual acuity is abbreviated VA and SE is the spherical equivalent refraction after six month.
(XLSX)

S2 File Acrysof data. The biometric information for patients who got the Acrysof SN60WF implanted. Right eyes are indicated with OD, left eyes with OS. The table shows the steep and flat keratometry values (K1, K2) their angles, the anterior chamber depth (ACD), the lens thickness (LT), the power of the implanted IOL (lens power), target refraction (ZR) and the refraction (in diopters) six month after surgery along with the spherical equivalent refraction (SE_6m).
(XLSX)
